# Efficacy of vertebral cryoablation and immunotherapy in a patient with metastatic renal cell carcinoma: a case report

**DOI:** 10.1186/s13256-019-2049-0

**Published:** 2019-04-21

**Authors:** Apiruk Sangsin, Hideki Murakami, Takaki Shimizu, Satoshi Kato, Hiroyuki Tsuchiya

**Affiliations:** Department of Orthopedic Surgery, Graduate School of Medical Sciences, Kanazawa University, 13-1 Takara-machi, Kanazawa, 920-8641 Japan

**Keywords:** Metastatic renal cell carcinoma, Vertebral cryoablation, Immunotherapy

## Abstract

**Background:**

In metastatic renal cell carcinoma, immunotherapy is the only treatment modality associated with a complete and durable response, but severe toxicity limits its usefulness. If toxicity could be eliminated, immunotherapy might be an effective treatment for metastatic renal cell carcinoma. We present a case of a patient with spinal metastatic renal cell carcinoma treated with total *en bloc* spondylectomy and reconstruction using a cryo-treated tumor-bearing bone graft; the patient demonstrated an antitumor cryoimmunological response.

**Case presentation:**

A 51-year-old Japanese man presented with back pain 4 years after undergoing a left-sided total nephrectomy for renal cell carcinoma. He was diagnosed with metastases in the T1–T3 vertebrae, right adrenal gland, sternum, left clavicle, and sacrum. Total *en bloc* spondylectomy and reconstruction using a cryo-treated tumor-bearing bone graft was performed to treat the vertebral metastases. Sunitinib and then everolimus were also administered. Serum interferon-γ and interleukin 12 levels were measured before surgery and at 1, 3, 6, and 12 months after surgery. Serum interferon-γ and interleukin 12 levels increased 3 months after surgery; this increase was sustained for 6 months. No local recurrence or other distant metastases occurred. The bone metastases remained stable, and the adrenal metastasis progressed slowly. The duration of progression-free survival during sunitinib and everolimus treatment was 24 and 40 months, respectively, and overall survival is currently 5.5 years.

**Conclusions:**

This report demonstrates that using cryo-treated tumor-bearing tissue in a patient with metastatic renal cell carcinoma stimulated an antitumor cryoimmunological response.

## Background

Renal cell carcinoma (RCC), a group of malignancies originating from the epithelium of renal tubules, is the most common type of kidney malignancy among adults. In 65% of cases, RCC is a localized disease that can be treated with total or partial nephrectomy; 35% of cases present with metastatic RCC (mRCC) [1]. Common sites of metastasis are the lungs, bones, lymph nodes, liver, adrenal glands,and brain[2].One-third of patients with mRCC have bone metastasis, most of which involves the spine [3]. In the era of molecular targeted therapy, improvements in median overall survival and progression-free survival have been observed; however, survival has been extended by only a few months [4].

Cryoablation, a method of cryotreatment, is a treatment option for RCC. RCC has shown what is called a cryoimmunological response *in vivo* [5]. However, there are very few clinical reports on this to date. We present a case of a 51-year-old man with mRCC involving multiple bones, including the T1–T3 vertebrae, who survived for more than 5 years after undergoing a total *en bloc* spondylectomy (TES) and reconstruction using a cryo-treated tumor-bearing bone graft.

## Case presentation

### History and clinical evaluation

**Fig. 1:**
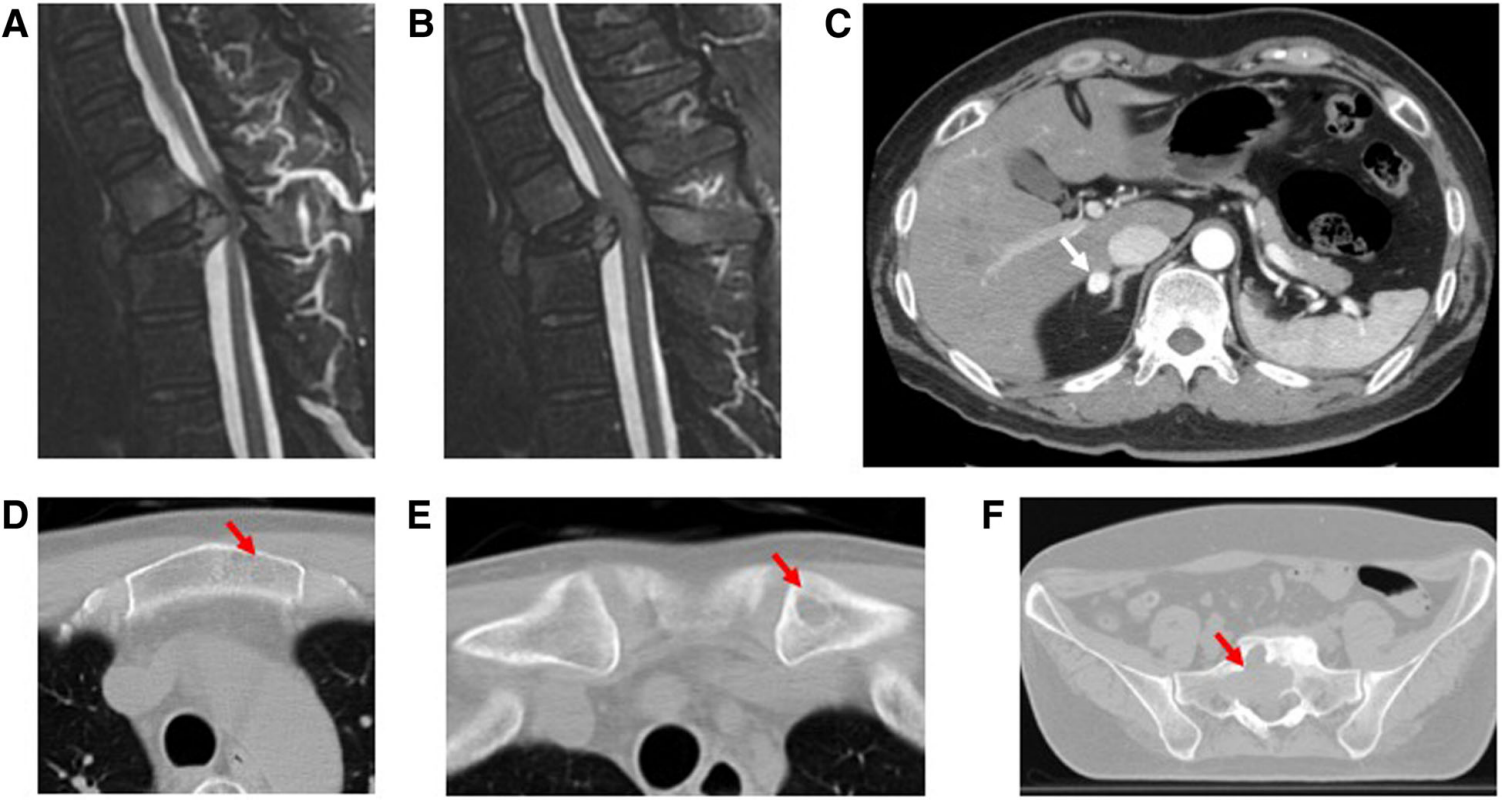
Sagittal magnetic resonance imaging scans demonstrate involvement of the T1–T3 vertebral bodies as well as T2 vertebral collapse (**a**) and tumor extension causing cord compression at the level of T2 (**b**). Axial computed tomographic images demonstrate metastases of the right adrenal gland (**c**), sternum (**d**), left clavicle (**e**), and sacrum (**f**) (*arrows*)

Our patient was a 51-year-old Japanese man who had undergone a left total nephrectomy for RCC 10 years ago. Four years later, he experienced back pain. Apart from sustained ankle clonus bilaterally, results of his physical examination were within normal limits. Magnetic resonance imaging and computed tomography (CT) of the spine revealed spinal metastases involving the T1–T3 vertebrae, with a pathological fracture of T2 causing spinal cord compression. Metastases were also detected in the right adrenal gland, sternum, left clavicle, and sacrum (Fig. 1). The pathology results of a CT-guided biopsy specimen of the T2 vertebral lesion were consistent with mRCC. Spinal metastases in this patient were classified as grade III according to Enneking classification, type 6 according to Tomita classification, and zones 4 to 9, layers A to D, according to Weinstein-Boriani-Biagini classification with a Spine Instability Neoplastic Score of 16, which indicated instability. The patient was treated with zoledronic acid 4 mg/month. One month after the diagnosis of spinal metastases, a TES with reconstruction—using a cryo-treated tumor-bearing bone graft—was performed.

### Surgery

TES was performed using a single posterior approach. The first, second, and third ribs were resected on both sides. The lower half of the C7 lamina was removed to expose the superior articular facet of T1. The posterior elements of the T1–T3 vertebrae were removed via pediculotomy using a flexible multifilament thread wire (T-saw; Pro Medical, Kanazawa, Japan). The cut surface of the pedicles was sealed with bone wax for hemostasis and to minimize tumor cell contamination due to the involvement of the T2 pedicles by the tumor. The T2–T3 nerve roots were ligated and cut bilaterally; the T1 nerve roots were preserved. Blunt dissection was performed around the T1–T3 vertebral bodies and C7/T1, T1/T2, T2/T3, and T3/T4 intervertebral discs. Bilateral pedicular screws were inserted and affixed to a rod from C7 to T5. An L-shaped chisel was used to cut through the C7/T1 intervertebral disc, and a T-saw was used to cut through the body of T3. The T1, T2, and upper half of the T3 vertebral bodies were removed *en bloc*. The tumor and soft tissues such as the ligaments, disc, and cartilage were removed from the excised tumor-bearing bone. The excised tumor-bearing bone was then immersed in liquid nitrogen at − 196 °C for 20 min, cut into small pieces, and packed into a titanium mesh cage. The cage then replaced the removed vertebrae, and, after being fixed to another rod, was slightly compressed by posterior instrumentation.

### Pathological findings

**Fig. 2:**
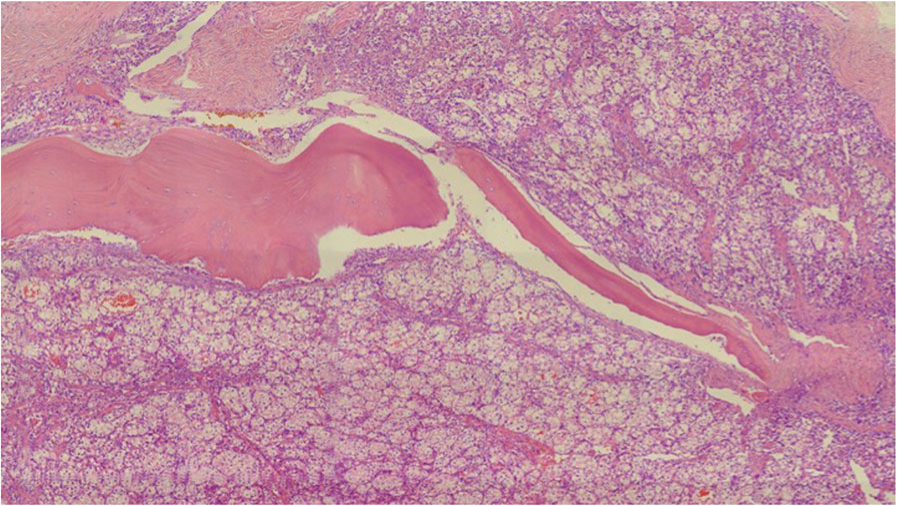
Microscopic features of the tumor from resected T1–T3 vertebral specimens showing compact, alveolar, and tubulocystic malignant cells with clear cytoplasm around the bone

The pathological findings of the affected vertebrae were consistent with a diagnosis of modified International Society of Urological Pathology grade 2 metastatic clear cell RCC (Fig. 2).

### Evaluation of antitumor cryoimmunology

Blood samples were collected from the patient before undergoing surgery and 1, 3, 6, and 12 months thereafter. Serum interferon (IFN)-γ and interleukin (IL)-12 concentrations were measured. The preoperative IFN-γ concentration was 133.0 IU/ml, and at 1, 3, 6, and 12 months after surgery, the concentrations were 79.4, 151.0, 145.0, and 42.0 IU/ml, respectively. The preoperative concentration of IL-12 was 60.4 pg/ml, and at 1, 3, 6, and 12 months after surgery, the concentrations were 53.1, 113.0, 107.0, and 62.2 pg/ml, respectively.

### Postoperative course

Sunitinib was started 3 months after surgery. This was changed to everolimus 2 years later because of a slight increase in size of the right adrenal gland metastasis from 15 mm to 17 mm. One month after TES, sacral radiotherapy was provided, with a total dosage of 45 Gy. Two years after the patient underwent TES, zoledronic acid was substituted with denosumab 120 mg/month because of slight progression of the sternal metastasis. Thereafter, the bone metastases remained stable.

**Fig. 3:**
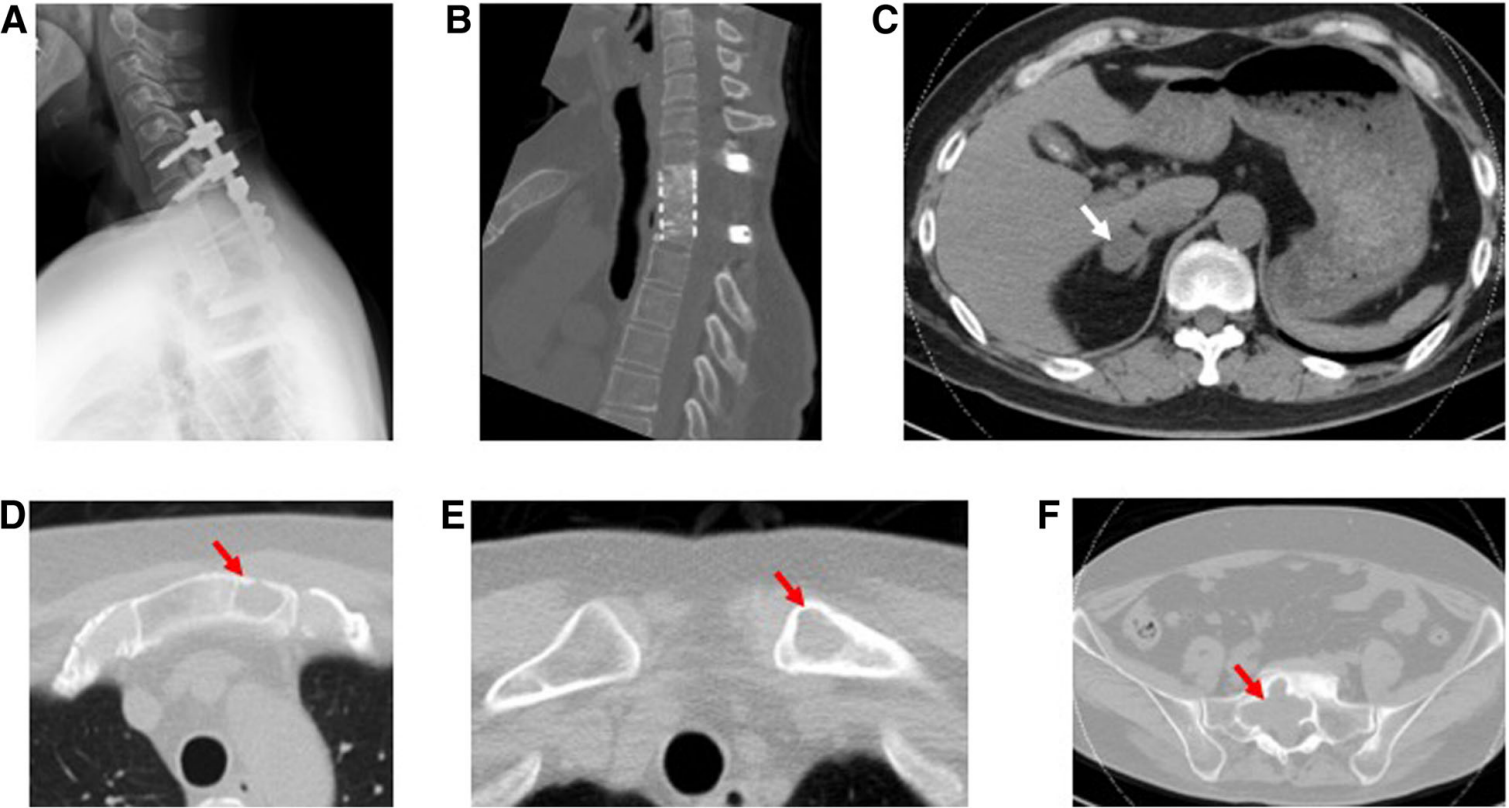
Postoperative imaging at follow-up, 5.5 years after total *en bloc* spondylectomy, showing well-maintained instrumentation (**a**) with radiographic bony union between adjacent vertebrae and the bone graft (**b**). There is no evidence of local recurrence. Axial computed tomographic images demonstrate the increasing size of the right adrenal gland metastasis (**c**) but stable metastases in the sternum (**d**), left clavicle (**e**), and sacrum (**f**) (*arrows*)

At the follow-up examination 6 months after TES, radiographic union between the bone graft site and the adjacent vertebrae had been achieved. At a recent follow-up appointment, 5.5 years after TES, no evidence of local recurrence at the spondylectomy site was demonstrated by CT (Fig. 3a, b). Moreover, the metastases in the sternum, left clavicle, and sacrum were stable. A sclerotic rim around the sacral lesion was clearly visualized (Fig. 3d, e, and f). The right adrenal gland metastasis gradually increased in size (to 24 mm) while the patient was receiving everolimus (Fig. 3c).

## Discussion

Before the era of targeted therapy, patients with mRCC survived for 10–22 months [6]. Since the emergence in 2005 of several targeted therapies for RCC, including kinase and checkpoint inhibitors, outcomes have improved median overall survival of patients who received first-line targeted therapy such as sunitinib [7]. Thus, TES may be considered in patients with spinal mRCC based on the suggestion that tumor excision should be done in patients with more than a 1-year predicted prognosis. A study by Kato *et al.* [8] showed that patients with spinal mRCC in the absence of liver metastasis could benefit from TES because liver metastasis is the only factor associated with short survival. In that study, 64% of patients with mRCC had multiple metastases but a median cancer-specific survival of 130 months, and a 69% 5-year survival rate was achieved after TES, both of which are more favorable than in other reports [9]. A possible reason for prolonged survival is that complete resection of spinal metastasis can prevent paralysis and intractable pain that severely compromise performance status of the patients. Moreover, cryo-treated tumor-bearing reconstruction after TES may produce an antitumor immune response [10, 11].

RCC is recognized as a tumor that is susceptible to immunotherapy, including IL-2 and recombinant IFN-α [12]. Although severe toxicity and side effects have been reported, this is the only treatment modality to which mRCC has shown a durable complete response [13]. Hence, if toxicity and side effects can be eliminated, immunological treatments remain promising for improving the survival of patients with mRCC.

Cryotreatment uses cold injury to kill tumor cells. It causes cellular dehydration and intracellular ice crystallization. After treatment, tumor-specific antigens remain intact and can stimulate immunological responses [11]. Higher concentrations of proinflammatory cytokines, including IL-1, IL-6, and nuclear factor-κB, are released after cryotreatment than after high temperature–based modalities [14]. Cryoablation, a method of cryotreatment, is a therapeutic option in RCC. It is a minimally invasive procedure that may stimulate cryoimmunological effects. In an *in vivo* study using renal cryoablation in an RCC murine model, a significant inflammatory response was observed in terms of neutrophil and macrophage infiltration into the treated renal parenchyma, blood vessels, and perivascular areas, and increased numbers of neutrophils, macrophages, and CD4^+^ and CD8^+^ T cells were observed. Moreover, an increased concentration of IFN-γ, a potent antitumor cytokine, was demonstrated [5]. However, clinical evidence regarding the usefulness of cryoimmunology in the treatment of RCC in humans is limited.

A cryoimmunological response has been shown in patients with tumors, including mRCC, who have received an autograft of cryo-treated tumor tissue (using liquid nitrogen) for surgical reconstruction. This method has long been used in our institution, both for structural bone grafts in limb reconstruction [15] and for morcellated bone grafts in TES [16]. Reconstruction using a structural autograft containing tumor treated by liquid nitrogen is now considered a standard method of reconstruction after extremity tumor excision [15]. Histological examination of structural frozen autograft treated by liquid nitrogen removed after implantation revealed that all tumor cells were eradicated from the frozen bone, and osteogenesis was observed in a broad portion of the bone [17]. In TES, no adverse effects were observed in all 56 patients who underwent reconstruction using a morcellated, cryo-treated, tumor-bearing bone graft with an average follow-up of 14 months. Although three patients had local recurrence, it occurred from tissue around the spinal column but not in the grafted bone [16]. In 2011, Nishida *et al.* reported increased serum levels of IFN-γ and IL-12 in patients who underwent reconstruction of malignant bone tumors using cryo-treated autografts. The level of IFN-γ increased by 155% and 268% at 1 and 3 months, respectively, compared with preoperative concentrations, whereas the concentration of IL-12 increased by 190% and 432% at 1 and 3 months, respectively. The serum IFN-γ and IL-12 concentrations in our patient presented in this report increased 1 month postoperatively, whereas a patient in a different report demonstrated continuous disease-free survival [18]. A case report by Nishida [19] demonstrated a clinical response to cryoimmunology in the distal femur and lungs of a patient with mRCC who underwent wide excision and reconstruction using a cryo-treated tumor-bearing structural autograft. Disappearance of the lung metastases was evident on a chest CT scan 10 months after surgery; this was accompanied by increased concentrations of serum IFN-γ and IL-12 at 1 and 3 months postoperatively, without other adjuvant therapy [19]. Our patient underwent TES and reconstruction with a cryo-treated tumor-bearing morcellated bone graft; this method has been used in our institution since 2010. With this method, the mean serum IFN-γ and IL-12 concentrations also increased significantly 1 and 3 months after surgery in a case series of 60 patients with spinal metastases [10]. In our patient, serum IFN-γ and IL-12 levels increased 3 months after surgery and were sustained for 6 months for IFN-γ and 12 months for IL-12. The increased concentrations of antitumor cytokines indicate that cryo-treated tumor tissues can induce immunological antitumor activation. To the best of our knowledge, our patient is the first with mRCC to demonstrate clinical systemic antitumor cryoimmunological effects after TES and reconstruction using a cryo-treated tumor-bearing morcellated autograft. There was no local recurrence at the spondylectomy site. Moreover, the combination of targeted therapies and systemic antitumor effects (possibly induced by the cryo-treated tumor tissue) resulted in a survival duration exceeding 5 years after diagnosis of mRCC. This is much longer than the median survival of patients treated with targeted therapies: A median overall survival of 34.85 months can be achieved using sunitinib-everolimus sequential treatment [20]. Although there was some progression of the right adrenal gland metastasis while our patient was receiving sunitinib and everolimus, the duration of progression-free survival was much longer in our patient than in patients treated with either sunitinib (24 vs 13.98 months) or everolimus (40 vs 5.56 months) alone [20]. Our patient did not carry any favorable prognostic factors, such as sunitinib-induced hypothyroidism and hypertension, that may lead to long survival [20], so prolonged survival in our patient may have resulted from induced antitumor cryoimmunological effects.

The present case report not only emphasizes the clinically proven efficacy of TES and reconstruction using cryoablation-treated tumor-bearing bone grafts in terms of local tumor control but also provides insight into cryotreatment as a potential option for mRCC. Cryotreatment could stimulate systemic endogenous cytokines that preserve effective antitumor properties while eliminating some of the serious complications that restrict the use of exogenous cytokines.

## Conclusions

Given its efficacy in our patient, feasibility studies of vertebral cryoablation in the context of mRCC should be conducted in large series. The presence of multiple metastatic sites should not stop the use of immunotherapy.

## Abbreviations

CT: Computed tomography
IFN-γ: Interferon-γ
IL: Interleukin
mRCC: Metastatic renal cell carcinoma
RCC: Renal cell carcinoma
TES: Total *en bloc* spondylectomy
